# Nationwide Representative Survey of Dietary Iodine Intake and Urinary Excretion in Postpartum Korean Women

**DOI:** 10.3390/nu13113955

**Published:** 2021-11-05

**Authors:** Do-Kyung Lee, Hunjoo Lee, Hyeyoung Lee, Taehyung Yoon, Seon-Joo Park, Hae-Jeung Lee

**Affiliations:** 1 Department of Health Sciences and Technology, GAIHST, Gachon University, Incheon 21999, Korea; boknyu88@naver.com; 2 Department of Integrated Risk Assessment Research, Chem.I.Net Ltd., Seoul 07964, Korea; adstar@cheminet.kr; 3 Nutrition and Functional Food Research Division, National Institute of Food and Drug Safety Evaluation, Cheongju 28159, Korea; leehy96@korea.kr (H.L.); thykfda@korea.kr (T.Y.); 4 Department of Food and Nutrition, College of BioNano Technology, Gachon University, Gyeonggi 13120, Korea; 5 Institute for Aging and Clinical Nutrition Research, Gachon University, Gyeonggi 13120, Korea

**Keywords:** dietary iodine, urinary iodine, postpartum women, *miyeokguk*

## Abstract

Iodine is an essential component of thyroid hormones, but excessive iodine intake can lead to thyroid dysfunction. Traditionally, Korean mothers consume brown seaweed soup (*miyeokguk*), a high source of iodine, after childbirth. There is controversy regarding the effects of excessive postpartum iodine intake on the health of mothers and infants. Thus far, there have been no nationwide large-scale surveys regarding the status of iodine intake among postpartum women in Korea. Therefore, we conducted a nationwide survey of postpartum dietary iodine intake among Korean women. In total, 1054 Korean women aged ≥19 years, at less than 8 weeks postpartum, participated in this survey. Dietary data were collected using self-reported 2-day dietary records, along with before-and-after meal photos. To evaluate the correlation between dietary iodine and urinary iodine excretion (UIE), spot urine, and 24 h urine samples were collected from 98 and 29 participants, respectively. The mean daily iodine intake among all participants was 2945.6 μg, and it gradually decreased over time after childbirth. Dietary iodine intake was significantly correlated with 24 h UIE (r = 0.396, *p* < 0.05) and spot urine UIE (r = 0.312, *p* < 0.05). Follow-up studies are required to examine the influence of excessive postpartum iodine intake on thyroid health in mothers and their infants.

## 1. Introduction

Iodine is an essential component of thyroid hormones, such as triiodothyronine (T3) and thyroxine (T4) [1]. Both T3 and T4 are essential for nerve and somatic cell development, and they have vital roles in early growth and development [2]. However, excessive or insufficient iodine intake can lead to thyroid disease [3]. A lack of iodine increases the size and number of thyroid epithelial cells, leading to enlargement of the thyroid gland [4]. Endemic goiter is a disease observed in iodine-deficient regions, such as several mountainous areas of Italy, Turkey, and Papua New Guinea [5,6]. Iodine deficiency is more prevalent in women than in men, and it occurs more frequently during pregnancy [7]. In pregnant women, iodine deficiency may lead to hypothyroidism [1], maternal goiter [8], and damage to the developing brains of the children [7]. Conversely, excessive maternal iodine intake can lead to thyroiditis, thyroidoma, and hyperthyroidism in neonates and infants [9]. Studies conducted in China have shown that excessive iodine intake increases the prevalence of thyroid dysfunction, autoimmune thyroiditis, and hypothyroidism [3,10]. Excessive iodine intake also increases the risk of papillary thyroid cancer [11].

Koreans consume more seaweed than do people from Western countries because of dietary cultural reasons and geographic location [12,13]. Seaweed, which is rich in iodine, is a popular side dish in Korean cuisine [14]. Ancient books from the Goryeo Dynasty (918–1392) and the Joseon Dynasty (1292–1910) recorded the tradition of women eating brown seaweed soup (*miyeokguk*) after childbirth [15]. In modern times, postpartum Korean women consume *miyeokguk* three times a day in the early postpartum period [16]. Rhee et al. [17] reported that a bowl of brown seaweed soup (250 mL) contains approximately 1705 μg of iodine. Based on this value, ingestion of brown seaweed soup three times daily would result in an estimated 5115 μg of iodine intake per day. However, the World Health Organization recommends 250 μg of iodine intake per day for pregnant and lactating women, with an upper limit of 500 μg/day [18]. Australia’s New South Wales government has warned Asian pregnant and lactating women that a high intake of iodine-rich seaweed soup may cause hypothyroidism in their children [19].

The effects of excessive iodine intake on the thyroid health of Korean mothers and infants are not fully known. A study of postpartum women in Korea found that high iodine intake from *miyeokguk* after childbirth was not associated with postpartum thyroiditis [8]. However, another study suggested that the high iodine content of breast milk may increase the risk of subclinical hypothyroidism in premature infants [20]. Most studies of the association between iodine intake and thyroid diseases in postpartum Korean women and infants have been conducted in small study populations or in only a few areas. Therefore, a nationwide large prospective study is required regarding the association between iodine intake and thyroid diseases in Korea.

However, there are no representative survey data on iodine intake in Korean postpartum women so far. Although the Korean National Health and Nutrition Examination Survey collects dietary data from postpartum women, few such participants have been included in the survey (<100 participants each year). Thus, this study was conducted to investigate iodine consumption among postpartum Korean women through a representative nationwide survey and to examine correlations between iodine intake and urinary excretion.

## 2. Materials and Methods

### 2.1. Participants

The study design of our research is a cross-sectional, observational study for postpartum women in Korea. Study participants were recruited from among women aged ≥19 years within 8 weeks of childbirth between May 2019 and September 2019, all of whom voluntarily consented to participate in the study. According to the G-Power program [21], a total sample size of 1052 was required for analysis of variance (ANOVA) comparisons between four groups with a significance level of 0.05, power of 0.99, and effect size of 0.15. Considering the characteristics of the postpartum women, the dropout rate was estimated to be approximately 30%.

As Korean mothers consume *miyeokguk* frequently (2–3 times daily) during the 2 months after childbirth, mothers within 8 weeks after delivery were selected as participants. Mothers with twin births and history of hyperthyroidism were excluded. For nationally representative sampling, samples were collected from among the five regions (Seoul Metropolitan Area, Yeongnam Area, Chungcheong Area, Gangwon Area, and Honam Area) using a proportional stratified sampling method based on the distribution of newborn infants reported by the National Statistical Information Service in 2017 [22]. The numbers of participants were assigned according to the postpartum period (1–2 weeks, 3–4 weeks, 5–6 weeks, and 7–8 weeks). The participants provided researchers with information regarding their place of residence and date of childbirth. All recruitment processes were conducted under the supervision of two experts.

As face-to-face interviews were not possible due to the limited external activities of the mothers and the high risk of infection of the infants, the researchers used a web-based questionnaire to collect general information and 2-day non-continuous dietary records. The researchers reviewed the completeness of the uploaded data and called participants for confirmation in the event of missing information. Among 1578 total participants, dropouts (*n* = 62), women who did not complete a 2-day diet record (*n* = 172), and women who did not submit before-and-after meal photos and food intake information (*n* = 290) were excluded. Finally, data from 1054 participants were used for the final analysis. All survey data were stored on a restricted-access computer. The collected survey data were anonymized and analyzed. This study was approved by the Gachon University Institutional Review Board (IRB No. 1044396-201901-HR-019-04). All participants provided informed consent prior to participating in the project.

### 2.2. Methods

#### 2.2.1. Survey Questionnaire

The questionnaire consisted of anthropometric data (height and weight), childbirth-related data (normal delivery, cesarean section, gestational age, newborn baby length, and newborn baby weight), feeding type (breastfeeding, formula feeding, and mixed feeding), subjective health status, health behaviors (drinking, smoking, and physical activity), household income level, history of chronic disease, dietary supplementation (without/containing iodine), beverage or water intake (e.g., coffee, tea), and *miyeokguk* intake. The level of physical activity was regarded as light because of the mothers’ postpartum statuses.

#### 2.2.2. Nutrient and Iodine Intake Assessments

A non-continuous 2-day dietary record was collected using a webpage to calculate the participants’ nutrient and iodine intake levels. Participants uploaded information regarding meals and snacks that they consumed over 2 days with before-and-after meal photos. The researchers monitored dietary records as the data were uploaded in real time. When food intake information described by a participant did not match the corresponding photos, the researchers checked the food intake by telephone.

Daily nutrient and iodine intake data were analyzed using the Computer-Aided Nutrition Analysis Program (Can-Pro) 5.0 (Korean Nutrition Society, Seoul, Korea). Iodine contents of foods for which the program did not have data were estimated based on (in order of priority) the Ministry of Food and Drug Safety 2015 analysis report [23], the Ministry of Food and Drug Safety 2012 analysis report [24], or Food composition tables version 9.1 of the Korean National Institute of Agriculture Science [25]. The revised database was reviewed by experts and used for the study. Iodine contents of dried brown seaweed and kelp, which contain the most iodine, were based on the Ministry of Food and Drug Safety 2015 analysis report [23] (dried brown seaweed, 15,800 μg/100 g; dried kelp, 192,700 μg/100 g). To calculate the dietary iodine intake per day, the amounts of iodine intake of dietary supplements were also added to the dietary iodine intake.

#### 2.2.3. Measurement of Urinary Iodine Concentration

To examine the correlation between dietary iodine intake and urinary iodine excretion (UIE), 98 postpartum women were recruited <4 weeks after childbirth to collect spot urine. In addition, 24 h urine samples were collected from 29 postpartum women in accordance with a protocol in which participants were asked to collect urine from the day after consuming two or more servings of *miyeokguk*; the collection times and volumes of urine samples were recorded. The collected samples were stored in a freezer and analyzed by inductively coupled plasma mass spectrometry (ICP–MS). As the analysis equipment, ICP–MS (Agilent 7800 Series, Agilent Technologies Inc., Santa Clara, CA, USA) was used. Iodide (1000 ppm in H_2_O, SPEX CertiPrep, Metuchen, NJ, USA) was used as standard material, and Rhodium (1000 ppm in 10% HCl, SPEX CertiPrep, Metuchen, NJ, USA) was used as an internal standard material. Quantification of iodine was carried out on the mass of the main isotope (I-127 *m*/*z*) of the element iodine. The limit of quantification (LOQ) was 0.475 µg/L, and the linearity was excellent up to 1000 µg/L (r = 0.999). To investigate the precision of the ICP–MS method, G-EQUAS quality two urine were analyzed in triplicate in two independent runs on different days within five days, and coefficient variation (CV%) was 1.1–2.0%. We used quality control samples with two levels of iodine concentrations (68.6 μg/L and 222.2 μg/L) provided by the German External Quality Assessment Scheme (G-EQUAS). As for the analysis method, precision, accuracy, linearity, and carryover (%) were evaluated based on the analysis method of the Centers for Disease Control and Prevention (CDC).

#### 2.2.4. Statistical Analysis

The participant characteristics are shown as the mean and standard error for continuous variables and percentage for categorical variables. The differences in general characteristics, anthropometric measurements, *miyeokguk* intake, and iodine intake were compared among the four groups of participants classified according to the postpartum period (1–2 weeks, 3–4 weeks, 5–6 weeks, and 7–8 weeks) by chi-squared test or one-way ANOVA with Duncan’s multi-range test. Pearson’s correlation analysis was used to examine the correlations of iodine intake with UIE in spot urine and 24 h urine samples. Data were analyzed using SAS version 9.4 (SAS Institute, Cary, NC, USA), and *p* < 0.05 was considered to indicate statistical significance.

## 3. Results

### 3.1. General Characteristics of Participants

The participants were divided into four groups according to postpartum period: 1–2 weeks, 368 (34.9%); 3–4 weeks, 351 (33.3%); 5–6 weeks, 201 (19.1%); and 7–8 weeks, 134 (12.7%). The regions of residence of the participants were divided into five areas: Seoul Metropolitan Area, Yeongnam Area, Chungcheong Area, Gangwon Area, and Honam Area. The distributions of the postpartum period and region are shown in Table 1.

The general characteristics of participants according to the postpartum period are shown in Table 2. There were no significant differences in age, height, or weight among groups. However, there were significant differences among groups in terms of the gestation period, feeding type, drinking status, and physical exercise.

The ratio of mixed feeding was high in the 1–2 weeks postpartum group because of the mothers’ physical recovery period; the ratio of mixed feeding was reduced and breastfeeding was significantly increased in the 3–4 weeks postpartum group, compared with the 1–2 weeks postpartum group. As 75% of mothers used postpartum care centers after childbirth [26] and exercised in accordance with the programs of these postpartum care centers, the level of physical activity was generally high at 1–2 weeks postpartum but decreased in subsequent postpartum periods.

### 3.2. Miyeokguk Intake

**Figure 1:**
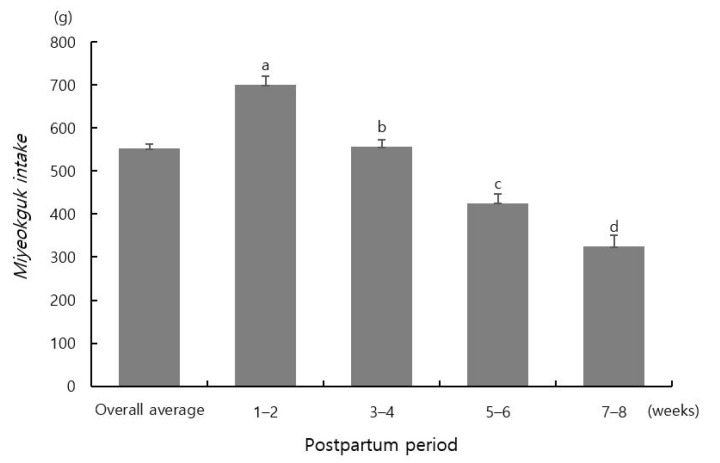
*Miyeokguk* consumption according to postpartum period. Different letters indicate significant differences in mean values among groups (*p* < 0.05, one-way ANOVA with Duncan’s multi-range test).

The mean daily intake of *miyeokguk* significantly differed among groups according to the postpartum period (*p* < 0.05) (Figure 1). Postpartum women consumed 700.3 ± 19.4 g of *miyeokguk* at 1–2 weeks, 555.7 ± 17.1 g at 3–4 weeks, 425.2 ± 21.5 g at 5–6 weeks, and 324.5 ± 26.1 g at 7–8 weeks on average.

### 3.3. Nutrient and Iodine Intake

The intakes of most nutrients significantly differed among groups (*p* < 0.05) (Table 3). The mean energy intake in the total study population was 2074.8 ± 18.9 kcal. The mean caloric intake consumed by mothers in the 1–2 weeks postpartum group was 2403 kcal, which was slightly higher than the recommended energy intake for nursing mothers in Korea (2240 kcal). In contrast, the 5–6 weeks postpartum and 7–8 weeks postpartum groups consumed 1776 kcal and 1805 kcal, respectively.

**Figure 2:**
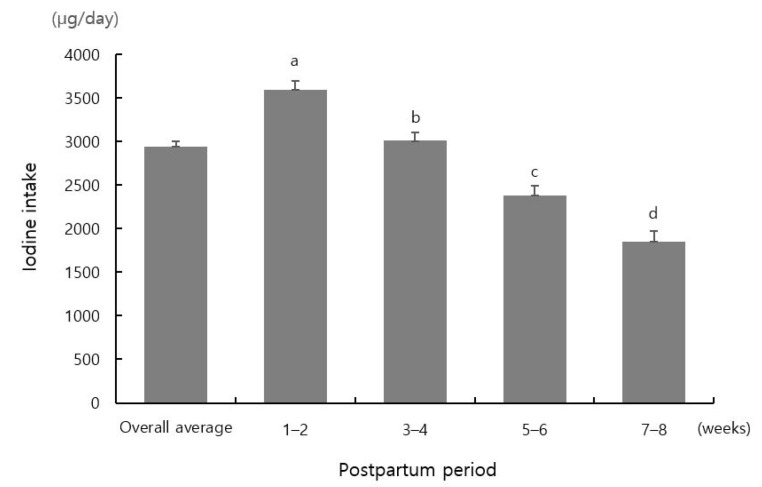
Mean daily iodine intake according to postpartum period. Different letters indicate significant differences in mean values among groups (*p* < 0.05, one-way ANOVA with Duncan’s multi-range test).

The mean daily iodine intake significantly differed among groups (*p* < 0.05) (Figure 2). The mean daily iodine intake of the total study population was 2945.6 μg. It gradually decreased over time after childbirth: 3597.0 μg at 1–2 weeks, 3008.0 μg at 3–4 weeks, 2376.9 μg at 5–6 weeks, and 1846.8 μg at 7–8 weeks postpartum on average.

### 3.4. Relation between Dietary Iodine Intake and Urinary Iodine Excretion

**Figure 3:**
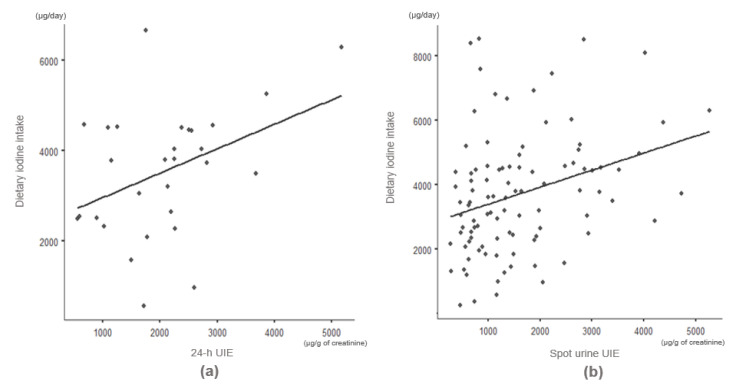
Correlations of dietary iodine intake with UIE in (**a**) 24 h urine samples (r = 0.396, *p* < 0.05) and (**b**) spot urine samples (r = 0.312, *p* < 0.05).

Dietary iodine intake was positively correlated with UIE in both 24 h urine samples (r = 0.396, *p* < 0.05) and spot urine samples (r = 0.312, *p* < 0.05) (Figure 3). Based on these correlation analysis results, linear regression analyses were conducted to determine whether iodine intake could be estimated from the UIE of 24 h or spot urine samples; the results showed that dietary iodine intake was significantly correlated with UIE in both spot urine samples (β = 0.312, *p* < 0.05) and 24 h urine samples (β = 0.290, *p* < 0.05).

## 4. Discussion

This study was conducted to assess the status of iodine intake in postpartum Korean women based on a sample of 1054 postpartum women across Korea. Traditionally, Korean mothers have substantially high levels of iodine intake in the early postpartum period because of *miyeokguk* consumption, which is presumed to help breast milk secretion and uterine contraction. Moon and Kim [16] reported that >90% of postpartum lactating Korean women consumed *miyeokguk* three times daily in the first postpartum week. According to the World Health Organization guidelines, the upper limit of daily iodine intake during lactation is 500 μg/day [18], and the Institute of Medicine recommends iodine intake levels of 220 μg/day during pregnancy and 290 μg/day during lactation, with an upper limit of 1100 μg/day for pregnant and lactating women [27]. In Korea, the upper limit of iodine intake for healthy adult men and women is set at 2400 µg, and there is no dietary iodine reference intake for pregnant or lactating women [28].

The mean daily intake of iodine was 2945.6 μg, and it decreased gradually over time after childbirth in this study. The maternal iodine intake was below the upper limit for adults (2400 μg) beginning at 5 weeks after delivery. Choue et al. [8] reported that iodine intake among Korean mothers during the first week after childbirth was 3366.6 μg/day; it decreased to 981.8 μg by 6 weeks postpartum. In another study of lactating women in Korea, the iodine intake was 2744 μg on postpartum days 2 and 5; it decreased to 1295 μg by 4 weeks postpartum [16]. Similarly, the results of the present study indicated that iodine intake among Korean mothers tended to gradually decrease after childbirth.

Our study showed a significant positive correlation between dietary iodine intake and UIE (24 h urine: r = 0.396, *p* < 0.05, spot urine: r = 0.312, *p* < 0.05). Similar to our results, a previous study of 40 lactating Korean women found that iodine intake was 3008 μg and UIE was 1070 μg/g (r = 0.368, *p* < 0.05) in the second week after delivery [29]. Another study of 137 Korean mothers demonstrated a positive correlation between iodine intake and UIE (r = 0.33, *p* = 0.001) [8]. Katagiri et al. [30] collected 24 h urine from 358 Japanese men and women and found that dietary iodine intake was significantly correlated with UIE (r = 0.37, *p* = 0.005). Although the level of iodine intake is significantly higher among Korean mothers than among nursing mothers in other countries, increased iodine intake by healthy mothers in the early postpartum period (1–2 weeks) is reportedly not associated with an increased incidence of thyroiditis [8,31]. Short-term excessive iodine intake can cause health problems in people with thyroid problems, while the Wolff–Chaikoff effect and sodium–iodine symporter can control excessive iodine intake in healthy people [32,33,34]. Most of the ingested iodine is excreted in the urine within 48 h [35]. Additionally, since most Koreans consume a lot of iodine from seaweed, the threshold for iodine intake may be genetically high; therefore, it seems that further research on this is necessary.

According to the analysis results of this study, many postpartum Korean women were taking dietary supplements. In 1054 postpartum women, we investigated whether they ingested dietary supplements and asked the product name, manufacturer, intake frequency, and single dose. Among the total subjects, 861 women (81.7%) answered that they were taking nutritional supplements after childbirth, and 107 women (12.4%) of them were taking dietary supplements containing iodine, such as multivitamins. The overall average iodine intake per day through nutritional supplements for 107 postpartum women taking nutritional supplements containing iodine was 181.6 μg. Since Korean mothers consume high amounts of iodine through seaweed soup, we think it is not necessary for them to take an iodine supplement.

This study had several strengths. First, to ensure representative nationwide sampling, data were collected by allocating participants based on the distribution of newborn infants published by the National Statistical Information Service in 2017. Second, to improve the accuracy of the dietary data, before-and-after meal photos were uploaded with information regarding meals and dietary supplements. There was a limitation in recruiting mothers who are willing to provide 24 h urine samples, so the number of participants in the 24 h urine excretion study was small. Therefore, further large-scale studies are needed to confirm the correlation between urinary and dietary iodine levels.

To our knowledge, this is the first nationwide survey to evaluate iodine intake status among postpartum Korean women. Our results can serve as basic data to establish dietary policy for public health improvement. Further follow-up studies are required to investigate the effects of excessive postpartum iodine intake on thyroid disease in mothers and their infants.

Ethics approval and consent to participate: Ethics approval for subject sampling was granted by the Gachon University Institutional Review Board (Reference Number 1044396-201901-HR-019-04).

## 5. Conclusions

There have been several studies on iodine intake in postpartum Korean women. However, these studies were either insufficient in the number of samples to become a nationwide representative sample or were limited to specific regions. In this study, the status of iodine intake was evaluated through a nationwide, representative survey of more than 1000 postpartum Korean women for the first time. The average daily iodine intake of postpartum Korean women was 2945.6 μg, and the iodine intake gradually decreased with the passage of time after childbirth. Subjects having high iodine intake showed high iodine excretion in 24 h and spot urine. Further follow-up study is necessary to investigate the causal relationship between high iodine intake of postpartum Korean women and thyroid diseases.

## Figures and Tables

**Table 1:** Distribution of participants according to region and postpartum period.

	Total (*n* = 1054)	1–2 Weeks (*n* = 368)	3–4 Weeks (*n* = 351)	5–6 Weeks (*n* = 201)	7–8 Weeks (*n* = 134)
Seoul	549 (52.2) ^(1)^	205 (55.7)	174 (49.6)	102 (50.7)	68 (50.7)
Yeongnam	255 (24.1)	95 (25.8)	84 (23.9)	40 (19.9)	36 (26.9)
Chungcheong	121 (11.5)	37 (10.1)	42 (12.0)	32 (15.9)	10 (7.5)
Gangwon	31 (2.9)	6 (1.6)	12 (3.4)	6 (3.0)	7 (5.2)
Honam	98 (9.3)	25 (6.8)	39 (11.1)	21 (10.4)	13 (9.7)

^(1)^ Values are presented as the number and percentage of participants; *n* (%).

**Table 2:** General characteristics of participants according to postpartum period.

Variables	Total (*n* = 1054)	1–2 Weeks (*n* = 368)	3–4 Weeks (*n* = 351)	5–6 Weeks (*n* = 201)	7–8 Weeks (*n* = 134)	*p*
Age (years)	32.5 ± 0.1 ^(1)^	32.4 ± 0.2	32.5 ± 0.2	32.2 ± 0.3	33.0 ± 0.3	0.231
Anthropometric data						
Height (cm)	161.9 ± 0.2	162.1 ± 0.3	161.9 ± 0.3	161.7 ± 0.4	161.5 ± 0.4	0.617
Weight (kg)	63.3 ± 0.3	63.6 ± 0.5	63.6 ± 0.5	62.6 ± 0.7	62.5 ± 0.8	0.447
Gestation period (weeks)	38.7 ± 0.1	38.9 ± 0.1	38.6 ± 0.1	38.6 ± 0.1	38.1 ± 0.2	<0.01
Delivery type						0.142
Normal delivery	532(50.5) ^(2)^	199 (54.1)	164 (46.7)	107 (53.2)	62 (46.3)	
Caesarean section	522 (49.5)	169 (45.9)	187 (53.3)	94 (46.8)	72 (53.7)	
Feeding type						<0.01
Breastfeeding	202 (19.2)	36 (9.8)	91 (25.9)	46 (22.9)	29 (21.6)	
Mixed feeding	762 (72.3)	322 (87.5)	234 (66.7)	129 (64.2)	77 (57.5)	
Formula feeding	90 (8.5)	10 (2.7)	26 (7.4)	26 (12.9)	28 (20.9)	
Alcohol drinking						<0.01
Yes	49 (4.6)	5 (1.4)	15 (4.3)	15 (7.5)	14 (10.4)	
No	1005 (95.4)	363 (98.6)	336 (95.7)	186 (92.5)	120 (89.6)	
Smoking						0.169
Current smoking	1 (0.1)	0 (0.0)	0 (0.0)	0 (0.0)	1 (0.7)	
Past smoking	151 (14.3)	45 (12.2)	53 (15.1)	31 (15.4)	22 (16.4)	
Non-smoking	902 (85.6)	323 (87.8)	298 (84.9)	170 (84.6)	111 (82.8)	
Regular physical exercise						<0.05
Yes	214 (20.3)	94 (25.5)	57 (16.2)	40 (19.9)	23 (17.2)	
No	840 (79.7)	274 (74.5)	294 (83.8)	161 (80.1)	111 (82.8)	
Monthly income (US $)						0.115
<2000	75 (7.1)	26 (7.1)	29 (8.3)	14 (7.0)	6 (4.5)	
2000–4000	597 (56.6)	192 (52.2)	208 (59.3)	114 (56.7)	83 (61.9)	
4000–6000	255 (24.2)	93 (25.3)	80 (22.8)	51 (25.4)	31 (23.1)	
>6000	127 (12.0)	57 (15.5)	34 (9.7)	22 (10.9)	14 (10.4)	
Chronic disease						0.633
Yes	67 (6.4)	20 (5.4)	26 (7.4)	11 (5.5)	10 (7.5)	
No	987 (93.6)	348 (94.6)	325 (92.6)	19 (94.5)	124 (92.5)	
Dietary supplement						0.535
Yes	861 (81.7)	297 (80.7)	295 (84.0)	163 (81.1)	106 (79.1)	
- Without iodine	754 (87.6)	260 (87.5)	256 (86.8)	141 (86.5)	97 (91.5)	
- Containing iodine	107 (12.4)	37 (12.5)	39 (13.2)	22 (13.5)	9 (8.5)	
No	193 (18.3)	71 (19.3)	56 (16.0)	38 (18.9)	28 (20.9)	

^(1)^ Values are presented as the mean ± standard error. ^(2)^ Values are presented as the number and percentage of subjects; *n* (%).

**Table 3:** Nutrient intake according to postpartum period.

	Total			1–2 Weeks			3–4 Weeks			5–6 Weeks			7–8 Weeks			*p*
	(*n* = 1054)			(*n* = 368)			(*n* = 351)			(*n* = 201)			(*n* = 134)			
Energy (kcal)	2074.8	±	18.9 ^(1)^	2403.8	±	30.8	2003.3	±	28.7	1776.7	±	35.6	1805.8	±	51.8	<0.01
Carbohydrate (g)	273.1	±	2.4	305.2	±	4.0	266.8	±	3.8	242.7	±	4.8	246.7	±	6.8	<0.01
Protein (g)	88.6	±	0.9	104.0	±	1.4	85.3	±	1.3	75.9	±	1.8	73.7	±	2.5	<0.01
Fat (g)	69.2	±	0.8	84.7	±	1.3	65.7	±	1.3	55.1	±	1.5	57.4	±	2.2	<0.01
Fibre (g)	37.8	±	0.4	45.1	±	0.7	37.3	±	0.7	31.1	±	0.9	28.6	±	1.3	<0.01
Vitamin A (RE)	579.2	±	9.1	706.7	±	14.8	537.5	±	14.2	476.0	±	19.1	492.7	±	28.0	<0.01
Retinol (μg)	146.7	±	3.9	154.2	±	5.2	137.4	±	6.3	135.1	±	9.3	167.6	±	15.9	0.034
Carotene (μg)	5190.0	±	95.1	6630.0	±	154.5	4801.5	±	151.1	4090.9	±	186.7	3901.5	±	272.1	<0.01
Vitamin C (mg)	78.4	±	1.4	97.7	±	2.3	74.6	±	2.2	63.7	±	2.8	57.8	±	3.2	<0.01
Vitamin B_1_ (mg)	1.9	±	0.02	2.1	±	0.03	1.8	±	0.03	1.6	±	0.04	1.6	±	0.06	<0.01
Vitamin B_2_ (mg)	1.8	±	0.02	2.2	±	0.04	1.7	±	0.03	1.6	±	0.04	1.6	±	0.06	<0.01
Niacin (mg)	15.7	±	0.2	18.7	±	0.3	15.0	±	0.3	13.3	±	0.3	13.2	±	0.5	<0.01
Calcium (mg)	672.2	±	8.5	756.5	±	12.7	668.0	±	15.2	597.8	±	19.0	563.7	±	23.0	<0.01
P (mg)	1311.0	±	13.5	1494.7	±	20.3	1284.0	±	23.8	1153.2	±	27.1	113.7	±	36.5	<0.01
Sodium (mg)	6156.4	±	68.2	7297.6	±	96.0	6048.3	±	110.8	5192.0	±	148.2	4752.4	±	182.0	<0.01
K (mg)	3757.2	±	40.6	4401.5	±	61.0	3727.5	±	63.8	3176.6	±	83.1	2936.6	±	115.2	<0.01
Fe (mg)	19.7	±	0.3	23.5	±	0.4	18.9	±	0.5	17.0	±	0.5	15.6	±	0.6	<0.01

^(1)^ Data are shown as the mean ± standard error.

## Data Availability

All data are reported in this manuscript.

## References

[1] Pérez-López F.R. (2007). Iodine and thyroid hormones during pregnancy and postpartum. Gynecol. Endocrinol..

[2] Wolmarans D.W. (2017). Maintaining euthyroidism: Fundamentals of thyroid hormone physiology, iodine metabolism and hypothyroidism. S. Afr. Fam. Pract..

[3] Teng W., Shan Z., Teng X., Guan H., Li Y., Teng D., Jin Y., Yu X., Fan C., Chong W. (2006). Effect of iodine intake on thyroid diseases in China. N. Engl. J. Med..

[4] Laurberg P., Cerqueira C., Ovesen L., Rasmussen L.B., Perrild H., Andersen S., Pedersen I.B., Carlé A. (2010). Iodine intake as a determinant of thyroid disorders in populations. Best Pract. Res. Clin. Endocrinol. Metab..

[5] Squatrito S., Delange F., Trimarchi F., Lisi E., Vigneri R. (1981). Endemic cretinism in Sicily. J. Endocrinol. Investig..

[6] Yordam N., Özön A., Alikaşifoğlu A., Özgen A., Ceren N., Zafer Y., Şimşek E. (1999). Iodine deficiency in Turkey. Eur. J. Pediatrics.

[7] Skeaff S.A. (2011). Iodine deficiency in pregnancy: The effect on neurodevelopment in the child. Nutrients.

[8] Choue R., Yim J., Cho Y., Lee W. (1997). The effects of dietary iodine intake on the postpartum thyrioditis (PPT) manifestation. Korean J. Nutr..

[9] Pennington J. (1990). A review of iodine toxicity reports. J. Am. Diet. Assoc..

[10] Tan L., Sang Z., Shen J., Liu H., Chen W., Zhao N., Wei W., Zhang G., Zhang W. (2015). Prevalence of thyroid dysfunction with adequate and excessive iodine intake in Hebei Province, People’s Republic of China. Public Health Nutr..

[11] Guan H., Ji M., Bao R., Yu H., Wang Y., Hou P., Zhang Y., Shan Z., Teng W., Xing M. (2009). Association of high iodine intake with the T1799A BRAF mutation in papillary thyroid cancer. J. Clin. Endocrinol. Metab..

[12] Jorgensen A., O’Leary P., James I., Skeaff S., Sherriff J. (2016). Assessment of breast milk iodine concentrations in lactating women in Western Australia. Nutrients.

[13] Mulrine H.M., Skeaff S.A., Ferguson E.L., Gray A.R., Valeix P. (2010). Breast-milk iodine concentration declines over the first 6 mo postpartum in iodine-deficient women. Am. J. Clin. Nutr..

[14] Ko Y.M., Kwon Y.S., Park Y.K. (2017). An iodine database establishment and iodine intake in Korean adults: Based on the 1998~2014 Korea National Health and Nutrition Examination Survey. J. Nutr. Health.

[15] Seo K. (2006). Almanac of Seasonal Customs of China.

[16] Moon S., Kim J. (1999). Iodine content of human milk and dietary iodine intake of Korean lactating mothers. Int. J. Food Sci. Nutr..

[17] Rhee S.S., Braverman L.E., Pino S., He X., Pearce E.N. (2011). High iodine content of Korean seaweed soup: A health risk for lactating women and their infants?. Thyroid.

[18] Andersson M., De Benoist B., Delange F., Zupan J. (2007). Prevention and control of iodine deficiency in pregnant and lactating women and in children less than 2-years-old: Conclusions and recommendations of the Technical Consultation. Public Health Nutr..

[19] New South Wales Government Warning to Pregnant and Breastfeeding Women: Seaweed Soup. Northern Sydney Local Health District 2012. https://www.mhcs.health.nsw.gov.au/publications/9120.

[20] Chung H.R., Shin C.H., Yang S.W., Choi C.W., Kim B.I. (2009). Subclinical hypothyroidism in Korean preterm infants associated with high levels of iodine in breast milk. J. Clin. Endocrinol. Metab..

[21] Faul F., Erdfelder E., Lang A.-G., Buchner A. (2007). G* Power 3: A flexible statistical power analysis program for the social, behavioral, and biomedical sciences. Behav. Res. Methods.

[22] Korean Statistical Information Service (2018). National Vital Statistics Reports 2017.

[23] Park K.S., Yang J.Y., Kim S.H., Lee J.Y. (2015). Study of International Standards for Iodine in Seaweed.

[24] Kang T.S., Lee J.H., Leem D., Seo I.W., Lee Y.J., Yoon T.H., Lee J.H., Lee Y.J., Kim Y.J., Kim S.G. (2012). Monitoring of Iodine in Foods for Estimation of Dietary Intake.

[25] Rural Development Administration (2019). Korean Food Composition Table.

[26] Ministry of Health and Welfare (2019). Year Book on Postpartum Care Center 2019.

[27] Leung A.M., Pearce E.N., Braverman L.E. (2011). Iodine nutrition in pregnancy and lactation. Endocrinol. Metab. Clin..

[28] Ministry of Health and Welfare, The Korean Nutrition Society (2020). Dietary Reference Intakes for Koreans 2020.

[29] Kim H., Lee H.N., Ha J. (2019). Association high-iodine-containing seaweed soup consumption after birth and subclinical hypothyroidism in Korean women: Korea National Health and Nutrition Examination Survey IV (2013–2015). Int. J. Thyroidol..

[30] Katagiri R., Asakura K., Uechi K., Masayasu S., Sasaki S. (2016). Iodine Excretion in 24-hour Urine Collection and Its Dietary Determinants in Healthy Japanese Adults. J. Epidemiol..

[31] Kim W.B., Yim C.H., Park K.S., Moon B.S., Lee J.H., Jun H.W., Jin H.J., Kim S.Y., Cho B.Y., Lee H.G. (1998). The incidence of postpartum thyroiditis and effect of high iodine intake on it in Korean women. J. Korean Soc. Endocrinol..

[32] Wolff J., Chaikoff I.L. (1948). Plasma inorganic iodide as a homeostatic regulator of thyroid function. J. Biol. Chem..

[33] Eng P.H., Cardona G.R., Fang S.L., Previti M., Alex S., Carrasco N., Chin W.W., Braverman L.E. (1999). Escape from the acute Wolff-Chaikoff effect is associated with a decrease in thyroid sodium/iodide symporter messenger ribonucleic acid and protein. Endocrinology.

[34] Leung A.M., Braverman L.E. (2014). Consequences of excess iodine. Nat. Rev. Endocrinol..

[35] Jahreis G., Hausmann W., Kiessling G., Franke K., Leiterer M. (2001). Bioavailability of iodine from normal diets rich in dairy products-results of balance studies in women. Exp. Clin. Endocrinol. Diabetes.

